# Can “Model Projects of Need-Adapted Care” Reduce Involuntary Hospital Treatment and the Use of Coercive Measures?

**DOI:** 10.3389/fpsyt.2018.00168

**Published:** 2018-05-01

**Authors:** Alexandre Wullschleger, Jürgen Berg, Felix Bermpohl, Christiane Montag

**Affiliations:** Department of Psychiatry and Psychotherapy, Berlin Institute of Health, Charité – Universitätsmedizin Berlin, Corporate Member of Freie Universität Berlin, Humboldt-Universität zu Berlin, Berlin, Germany

**Keywords:** need-adapted care, assertive community treatment, intensive case management, hometreatment, outpatient care

## Abstract

Intensive outpatient models of need-adapted psychiatric care have been shown to reduce the length of hospital stays and to improve retention in care for people with severe mental illnesses. In contrast, evidence regarding the impact of such models on involuntary hospital treatment and other coercive measures in inpatient settings is still sparse, although these represent important indicators of the patients' wellbeing. In Germany, intensive models of care still have not been routinely implemented, and their effectiveness within the German psychiatric system is only studied in a few pioneering regions. An innovative model of flexible, assertive, need-adapted care established in Berlin, Germany, in 2014, treating unselected 14% of the catchment area's patients, was evaluated on the basis of routine clinical data. Records of *n* = 302 patients diagnosed with severe mental disorders, who had been hospitalized at least once during a 4-year-observational period, were analyzed in a retrospective individual mirror-image design, comparing the 2 years before and after inclusion in the model project regarding the time spent in hospital, the number and duration of involuntary hospital treatments and the use of direct coercive interventions like restraint or isolation. After inclusion to the project, patients spent significantly less time in hospital. Among patients treated on acute wards and patients with a diagnosis of psychosis, the number of patients subjected to provisional detention due to acute endangerment of self or others decreased significantly, as did the time spent under involuntary hospital treatment. The number of patients subjected to mechanical restraint, but not to isolation, on the ward decreased significantly, while the total number of coercive interventions remained unchanged. Findings suggest some potential of intensive models of need-adapted care to reduce coercive interventions in psychiatry. However, results must be substantiated by evidence from randomized-controlled trials and longer observation periods.

## Introduction

Although many efforts have been made in the past years, the use of coercive measures like involuntary hospital treatment, seclusion, mechanical restraint and forced medication in psychiatric inpatient setting still belongs to everyday practice. The experience of coercion has been shown to be linked to a wide range of negative consequences, from higher rates of subsequent involuntary admissions, deterioration of the therapeutic relationship, lower use of outpatient resources to the development of post-traumatic symptoms (1–3). It also negatively influences private relationships and future professional perspectives (4). Coercion also runs against most staff members' self-conception of their work and as such constitutes a high burden on their health and well-being (5). Interventions aiming at reducing the use of coercion in inpatient setting have been developed over the last years. They often include multiple strategies, e.g., staff training, use of advance directives and crisis plans, modification of ward environments, early evaluation and identification of risk situations, or changes in ward routines (6, 7).

In the meantime, outpatient care has undergone dramatic evolutions and many models have been designed and introduced to address the needs of the most severely ill patients, who are most frequently subjected to coercion in inpatient settings (8). These initiatives often comprise the building of outreaching multiprofessional teams and of flexible, intensive, comprehensive and long-term care offers, as in the well-known models of assertive community treatment (ACT), Intensive Case Management (ICM) or Flexible ACT (FACT), that has been developed in the Netherlands to address the specific needs of the most severely ill patients and offer them a flexible shift between different intensities of care on a need-orientated basis (9, 10). Beside these models providing long-term intensive care, other approaches aiming at delivering time-limited interventions such as Crisis Resolution Teams (CRT) (11) have been developed that provide intensive outpatient care with the goal of an early identification and management of crises to prevent further exacerbation and hospital admission.

In contrast, German in- and outpatient psychiatric services are still characterized by structural and conceptual fragmentation, as traditional reimbursement practices do not incentivize the integration of sectors and treatment settings. From 2014, legislation has provided for the development of “model projects” (so-called “Modellprojekte” according to §64b German Social Code V) in order to evaluate various concepts of need-adapted care that may help to overcome sector divisions. In less than 20 pioneering regions, financial resources can be shifted to the outpatient sector in order to adequately address the needs of patients and to reduce hospital stays. One of these model projects was implemented at the Department of Psychiatry of the Charité at the St. Hedwig Hospital (PUK SHK) in 2014.

While some evidence supports the positive effects of intensive models of community care on patients' time spent in hospital, housing stability and on retention in care (8), data regarding their impact on involuntary hospital treatments and on the use of coercive measures like seclusion and restraint are not conclusive. One could expect, on the one hand, that the provision of comprehensive, outreaching care could improve treatment adherence, prevent crises and avoid severe deteriorations, which otherwise might set the stage for the use of coercion. On the other hand, intensive treatment models might attract severely ill patients with a higher risk for experiencing coercive interventions (12).

In a RCT comparing the implementation of CRT with CMHT, Johnson et al. showed that CRT did not have a significant impact on involuntary detentions (13). Similarly, the Danish OPUS trial showed in a comparison of ACT with standard community treatment no statistically significant differences in the use of coercion (14). Two other studies even showed that CRT/ACT led to a relative increase of compulsory hospital admissions (15, 16). Both authors argue that CRT/ACT might be efficient in reducing voluntary admissions by early crisis management, but not in preventing the deterioration of those service users' health, who could not be sufficiently engaged in treatment. Moreover, the British REACT study did not substantiate reductions of involuntary hospital admissions, when ACT was compared to standard treatment by community mental health teams. Of note, rates of involuntary admissions and criminal outcomes remained similar in both groups, though ACT patients were characterized by more complex needs and were treated more often under community treatment orders (17). However, model fidelity might be the most decisive factor regarding the effects of a treatment structure (18). In Germany, the analysis of the effects of an integrated care model designed for patients suffering from psychoses introduced in Hamburg showed a significant reduction of the number of involuntary hospital admissions after 2 and 4 years (19, 20). Another study regarding the effects of German regional psychiatric budgets (RPB)—another German exemplary financing model of psychiatric care—established that RPBs led to an increase in the rate of voluntary admissions, as well as to a decrease of the average length of stay and the use of mechanical restraint in hospital (21). Hence, there is actually no international consensus on the effects of such models on the use of detention and coercive measures. Moreover, the considerable differences between national legislations do not allow the generalization of study results and thus underline the need for studies adapted to particular national contexts.

The present study aims at evaluating the effect of a “model project of need-adapted care”, established at the Department of Psychiatry of the Charité at the St. Hedwig Hospital (PUK SHK) in Berlin in 2014, on the number of days spent in hospital, the necessity of involuntary hospital treatment and the frequency of use of coercive measures. Involuntary hospital treatment was defined through (1) provisional detentions, (2) detentions by court order according to Mental Health Law (Berlin PsychKG) or (3) detentions initiated by the patients' legal guardians, followed by court order according to German Civil Code. Coercive measures were defined as (1) mechanical restraint (fixation)—always combined with forced medication—and (2) isolation. To achieve this goal, an unselected sample of all patients, who had been automatically provided with the new treatment option from 2014 and hospitalized at least once during the 4-year observation period, was examined. In a retrospective design, routine hospital data on service use, involuntary hospital treatment and the application of coercive interventions were compared between the 2 years before and 2 years after implementation of the model project. It was hypothesized that entering the integrated treatment model would be associated with reductions of time spent in hospital and the experience of involuntary hospital treatment and coercive interventions.

## Methods

### Description of treatment

**Figure 1:**
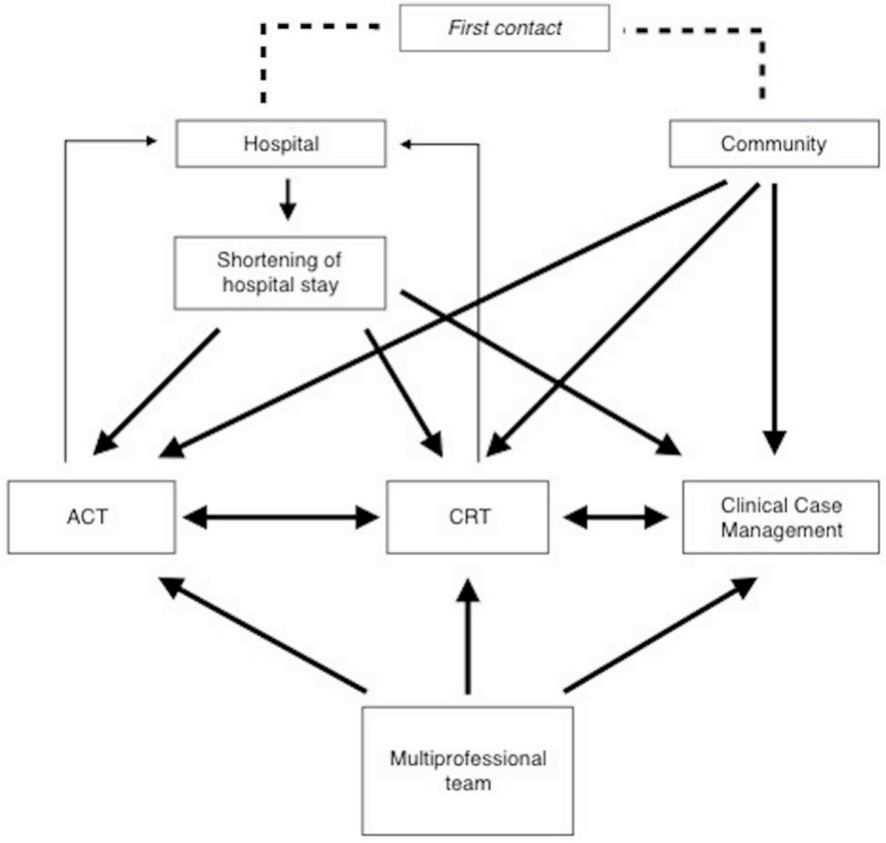
Description of the model project of need-adapted care. ACT, Treatment modus comparable to Assertive Community Treatment; CRT, Treatment modus comparable to Crisis Resolution Team.

A “model project of need-adapted care” (Modellprojekt according to §64b German Social Insurance Code (SGB-V) was introduced in 2014 at the PUK SHK and builds on an earlier pilot project of hometreatment for severely ill patients. It is based on a cooperation between the hospital and an insurance company, all of whose insured patients are automatically included in the project (about 14% of all in- and outpatients treated by the hospital). As in ACT, the core of the model is constituted by a specialized team, including psychiatrists, psychiatric nurses, a psychologist, a social worker, occupational and art therapists, that collectively bears the responsibility for patients and aims at a high personal and conceptual continuity of care. As in ACT, treatment is not limited to acute crises and the model also allows for flexible, short and longer term multiprofessional outreach. A 24/7 phone availability is warranted. The provided support also encompasses the identification and management of social issues. As in FACT, a central role is played by the main therapist, who gathers information, acts as case manager and intermediary between all team members and regulates the intensity of care provided. This high flexibility allowing to shift between different intensity levels of care according to patients needs is a central component of the model project. The frequency and intensity of contacts of care are constantly modulated to adapt to the actual state of the patient, during crises or rehabilitation phases. Periods of clinical case management might thus alternate with periods of shared care if needed. However, due to a considerably higher case load per person and the case-mix including patients with psychotic and non-psychotic diagnoses, the model project only shows low fidelity to ACT, FACT or other well-established concepts; international comparability is therefore limited. The structure of the model project is depicted in Figure 1.

Strong focus is laid, however, on an open-dialog-based and psychotherapeutic approach as well as on the provision of specific treatment for patients with psychoses and/or first manifestations of a psychiatric illness. First contact with the team can be established directly in the outpatient and emergency setting or during an inpatient stay. The model aims at reducing the length of hospital stay for the benefit of a more intensive and sustainable treatment in the patients' own living environment. A further goal is the reduction of involuntary hospital admissions and detentions as well as of other coercive interventions.

### Design

**Figure 2:**
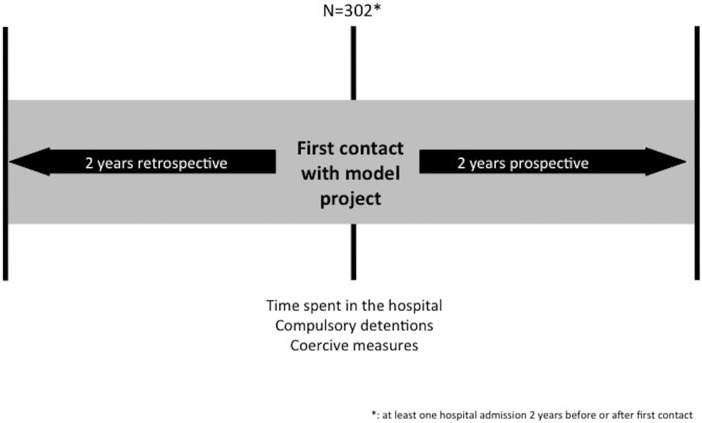
Study design.

The study used a pre- post-mirror comparison design. All patients in the model project who had been admitted to the hospital at least once during the 4-years studied period were included. Data about the lengths of stays, involuntary hospital treatment and the use of coercive measures 2 years before and up to 2 years after their first contact with the model project were retrieved from the clinic information system. This design is summarized in Figure 2.

Involuntary hospital treatment included (1) provisional detentions for 24 h maximum initiated by the borough office, the police president or legally mandated psychiatric hospitals (§23.1, §23.2 Berlin Mental Health Act/PsychKG, 2016), (2) detention by court order (§15 Berlin Mental Health Act/PsychKG, 2016), both assuming the presence of acute endangerment of self and others, and (3) detentions with the purpose of treatment initiated by a legal guardian according to §1906 German Civil Code (BGB). Fixation is defined as the use of mechanical restraint, which was in all cases combined with involuntary medication. Isolation is defined as seclusion in a locked room, both under permanent staff surveillance.

The study was conducted solely on the basis of anonymized routinely collected data that were retrieved from the hospital information system and did not imply the direct involvement of patients. Hence, it did not require the approval of the local ethics committee. Data quality can be considered high, as data on detentions and other coercive intervention are invariably gathered in the hospital records, completely available and must be reported to the Senate of Berlin.

### Sample

Overall, 302 Patients, who had been hospitalized at least once in the observation period, were included in the study. Among them, *n* = 193 were admitted to acute wards, where coercive measures can take place. A second subsample of patients with drug-induced, schizophrenia spectrum, manic or bipolar psychoses (F1x.5, F2x, F30, F31) was also considered for separate data analysis (*n* = 109), because the model project lays strong focus on this particular subgroup. The characteristics of included patients are described in Table 1.

**Table 1:** Sample characteristics.

	**All patients (*n* = 302)**	**Patients treated on acute wards (*n* = 193)**	**Patients with psychoses (F1x.5, F2x, F30, F31) (*n* = 109)**
**Sex**			
Female n (%)	146 (48.3%)	97 (50.3%)	55 (50.5%)
Male n (%)	156 (51.7%)	96 (49.7%)	54 (49.5%)
Age (mean ± SD) (years)	39.88 (±12.51)	40.03 (±12.82)	40.27 (±12.51)
Time in model project (mean ± SD) (months)	27.73 (±12.89)	28.87 (±12.67)	30.22 (±12.58)
**Diagnostic group n (%)**			
F0x	2 (0.7%)	1 (0.5%)	–
F1x	75 (24.8%)	17 (8.8%)	
F1x.5, F2x, F30, F31	109 (36.1%)	105 (54.4%)	
F3x unipolar, F4x	79 (26.2%)	48 (24.9%)	
F6x and others	37 (12.2%)	22 (11.4%)	

### Analysis

Data about the time spent in hospital for all 302 patients as well as the number and duration of involuntary hospital treatment and coercive measures regarding patients admitted to acute psychiatric wards and patients with diagnosis of psychoses were retrieved from the hospital information system and medical records. Previous experience of involuntary hospital treatment and coercive measures was used as a variable for analysis purposes. We performed a statistical analysis including calculation of means per 100 patient-days and pre- post-comparison of means using Wilcoxon signed-rank and McNemar tests using SPSS 24.0.

## Results

### Time spent in the hospital

The analysis showed that the inclusion in the model project led to a statistically significant reduction of the time spent in the hospital in the whole studied sample as well as in the groups of patients treated on acute wards and patients with psychoses. The results are summarized in Table 2.

**Table 2:** Number of days spent in the hospital before and after inclusion in the model project.

	**Time spent in hospital (pre-inclusion) (days/100 patient-days) mean (± SD)**	**Time spent in hospital (post-inclusion) (days/100 patient-days) mean (± SD)^††^**
Whole sample (*n* = 302)	4.03 (±6.16)	3.73 (±7.10)^*^
Acute wards patients (*n* = 193)	5.04 (±6.89)	4.03 (±7.79)^**^
F1x.5, F2x, F30, F31.x (*n* = 109)	6.18 (±8.04)	4.69 (±8.78)^*^

†† *Pre- post-comparison using Wilcoxon signed-rank test*.

SD, Standard deviation;

* p < 0.05;

** *p < 0.01*.

### Number and duration of involuntary hospital treatment

When considering patients treated on acute wards and patients with a diagnosis of psychosis, the number of persons subjected to provisional detention (max 24 h, initiated by police president or hospital psychiatrists) according to Mental Health Law (Berlin PsychKG) significantly decreased after their inclusion in the model project. The average number of provisional detentions also significantly decreased. These results are summarized in Table 3.

**Table 3:** Number of patients subjected to provisional detention and average number of provisional detentions before and after inclusion in the model project.

	**Patients subjected to provisional detention (pre-inclusion) n (%)**	**Patients subject to provisional detention (post-inclusion) n (%)^†^**	**Provisional detentions/100 patient-days (pre-inclusion) mean (±SD)**	**Provisional detentions/100 patient-days (post-inclusion) mean (±SD)^††^**
Acute wards patients (*n* = 193)	44 (22.8%)	17 (8.8%)^***^	0.04 (±0.11)	0.02 (±0.09)^**^
F1x.5, F2x, F30, F31.x (*n* = 109)	35 (32.1%)	13 (11.9%)^**^	0.06 (±0.13)	0.03 (±0.11)^**^

† Pre- post-comparison using McNemar test;

†† *pre- post-comparison using Wilcoxon signed-rank test*.

SD, Standard deviation;

** p < 0.01;

*** *p < 0.001*.

When considering patients who were at least once subjected to provisional detention in the 2 years preceding their inclusion in the model project (*n* = 49), the analysis also shows a statistically significant reduction in the average number of provisional detentions they experienced in the 2 following years (mean/100 patient-days ±SD) (pre: 0.19 ± 0.15; post: 0.04 ± 0.12) and the number of them experiencing such measures again (*n* = 6).

When analyzing the effects of the inclusion in the model project on detentions by court order according to Mental Health Law (Berlin PsychKG), results show that as in the case of provisional detentions, the number of patients subjected to this type of detention decreased in both subsamples, but did not reach statistical significance. However, the time spent under compulsory detention was significantly reduced in both subsamples (Table 4). In the subgroup of patients, who were at least once subject of compulsory detention in the 2 years preceding their first contact with the model project (*n* = 25), data show that a vast proportion of them did not suffer again such a measure in the time following their inclusion (*n* = 3). These patients were also shown to have spent less time under compulsory detention after inclusion (mean (days/100 patient-days) ±SD) pre: 2.56 ± 1.76; post: 0.59 ± 1.78). Both differences were statistically significant.

**Table 4:** Number of patients subject to compulsory detention and average duration of compulsory detention before and after inclusion in the model project.

	**Patients subjected to detention (pre-inclusion) n (%)**	**Patients subject to detention (post-inclusion) n (%)^†^**	**Time spent under detention (pre-inclusion) (days/100 patient-days) mean (± SD)**	**Time spent under detention (post-inclusion) (days/100 patient-days) mean (± SD)^††^**
Acute wards patients (*n* = 193)	22 (11.4%)	12 (6.2%)	0.31 (±1.04)	0.13 (±0.65)^*^
F1x.5, F2x, F30, F31.x (*n* = 109)	19 (17.4%)	9 (8.3%)	0.51 (±1.33)	0.19 (±0.82)^*^

† Pre- post-comparison using McNemar test;

†† *pre-post-comparison using Wilcoxon signed-rank test*.

SD, Standard deviation;

* *p < 0.05*.

When considering formal detentions initiated by the patients' legal guardians (Berlin BGB), the analysis of all patients' subsamples showed no reduction of the number of patients subjected to this type of detention or of the duration of detention (Table 5). Among the patients subjected to detention under guardianship 2 years before inclusion (*n* = 17), only 11.8% of them were legally detained under guardianship in the two following years (*n* = 2). This reduction was statistically significant.

**Table 5:** Number of patients subject to formal detention initiated by their legal guardian (German Civil Code) and average duration of detention before and after inclusion in the model project.

	**Patients subject to detention under guardianship (pre-inclusion) n (%)**	**Patients subject to detention under guardianship (post-inclusion) n (%)^†^**	**Time under detention under guardianship (pre-inclusion) (days/100 patient-days) mean (±SD)**	**Time under detention under guardianship (post-inclusion) (days/100 patient-days) mean (±SD)^††^**
Acute wards patients (*n* = 193)	12 (6.2%)	10 (5.2%)	0.64 (±3.23)	0.74 (±4.39)
F1x.5, F2x, F30, F31.x (n = 109)	10 (9.1%)	10 (9.1%)	1.07 (±4.22)	1.31 (±5.78)

† Pre- post-comparison using McNemar test;

†† *pre-post-comparison using Wilcoxon signed-rank test*.

*SD, Standard deviation*.

### Coercive interventions

The results show a decrease of the number of patients who experienced mechanical restraint during their hospital stay. This was shown to be statistically significant in the group of patients treated on acute wards, but not by patients with a diagnosis of psychosis. On the contrary, no statistically significant effect could be shown regarding the average number of restraints, the number of patients subjected to seclusion or the number of seclusive events (Tables 6, 7).

**Table 6:** Number of patients subject to seclusion or restraint before and after inclusion in the model project.

	**Restraint**		**Seclusion**	
	**Patients subject to restraint (pre-inclusion) n (%)**	**Patients subject to restraint (post-inclusion) n (%)^†^**	**Patients subject to seclusion (pre-inclusion) n (%)**	**Patients subject to seclusion (post-inclusion) n (%)^†^**
Acute wards patients (*n* = 193)	15 (7.7%)	6 (3.1%)^*^	16 (8.3%)	10 (5.2%)
F1x.5, F2x, F30, F31.x (*n* = 109)	12 (10.9%)	5 (4.5%)	15 (13.8%)	8 (7.3%)

† *Pre- post-comparison using McNemar test*.

* *p < 0.05*.

**Table 7:** Number of seclusions and restraints before and after inclusion in the model project.

	**Restraint**	**Seclusion**
	**Restraints/100 patient-days (pre-inclusion) mean (±SD)**	**Restraints/100 patient-days (post-inclusion) mean (±SD)^††^**	**Seclusions/100 patient-days (pre-inclusion) mean (±SD)**	**Seclusions/100 patient-days (post-inclusion) mean (±SD)^††^**
Acute wards patients (*n* = 193)	0.02 (±0.08)	0.01 (±0.06)	0.03 (±0.17)	0.04 (±0.25)
F1x.5, F2x, F30, F31.x (*n* = 109)	0.03 (±0.09)	0.02 (±0.08)	0.05 (±0.22)	0.07 (±0.32)

†† *Pre- post-comparison using Wilcoxon signed-rank test*.

*SD, Standard deviation*.

When data on restraints and seclusive events are combined, a statistically significant decrease of the number of persons subjected to coercive events on acute wards can be noted [pre-inclusion: 20 (10.4%); post-inclusion: 10 (5.2%)]. Among the subgroup of patients who experienced at least one coercive event (restraint or seclusion) (*n* = 23) in the 2 years preceding first contact with the model project, only 2 (8.7%) experienced another coercive event in the time following their inclusion (*p* < 0.05).

## Discussion

The analysis and comparison of routine hospital data on service use, involuntary hospital treatment and coercive interventions preceding and following the inclusion of patients to a “model project of need-adapted care” showed that this novel treatment approach led to a statistically significant reduction of patients' time spent in hospital. In our study, the subgroup of patients with a diagnosis of psychosis particularly benefited from the effects of the model project, thus indicating that one of its main goals, i.e., providing alternatives to hospital admissions for patients with SMI, could be attained. Many ICM/ACT models such as the integrated care model of the University of Hamburg already focus their resources on this patient subgroup in order to provide assertive treatment to a population which exhibits high rates of treatment drop-out, comorbid disorders and lower treatment adherence (20). Factors contributing to the positive effects of ACT/ICM have been analyzed in previous works. Notably, Burns and colleagues identified six characteristics of hometreatment models that were linked to a reduction of inpatient bed use: regular visits at home, a high proportion of contacts at home, smaller caseloads, responsibility for health and social care, multidisciplinary teams and a psychiatrist being integrated in the team (22). Many of these features are shared by the “model project of need-adapted care.”

However, lower use of inpatient facilities may not necessarily correspond to patients' wellbeing, and data on coercive interventions are certainly more useful indicators of patients' health and the quality of a therapeutic process. In this study, results also showed that after inclusion in the model project, fewer patients were subjected to provisional detentions under Mental Health Law and that the number of provisional detentions and the time spent under detention could be significantly reduced, whereas the use of detentions under guardianship could not be shown to be significantly influenced by the model project. This effect of intensive, flexible outpatient care on involuntary hospital treatment was only partly shown by previous studies (20, 21). Previous studies of flexible ACT models such as FACT that shares some similarities with our model project didn't investigate their effects on detentions (23). However, findings of our study are in keeping with results of treatment approaches with an exclusive focus on psychoses that also showed pronounced reductions in involuntary admissions in Germany (19).

Regarding the treatment model, the reduction of provisional detentions and detentions under Mental Health Law can at least partly be explained by the high level of flexibility in the intensity of care it provides. Through the work of outreaching, multiprofessional teams, crisis intervention may take place in the patients' domestic setting, but can also facilitate voluntary hospital admissions when necessary. However, other factors such as staff selection and enthusiasm in the development of an innovative treatment project must be considered.

Interestingly, a majority of patients, who experienced compulsory detentions before their inclusion in the model project, were not again subject of involuntary hospital treatment after their first contact with the team. Here, the provision of long-term assertive outpatient care with a high level of conceptual continuity, including the availability of individualized plans for the recognition and the containment of crises in their early stages, seems to particularly benefit those patients, whose course of disease led to recurring involuntary hospitalizations in the past. Moreover, clinical impression as well as scientific evidence advocate the importance of trust-building and the quality of the therapeutic relationship as decisive active ingredients in the treatment of patients with psychoses (24).The longer-term focus on the working relationship may specifically help to prevent involuntary hospital treatment. Hence, both the high flexibility of service provision and the long-ranging character of the model project may contribute to a reduction of involuntary hospital treatments.

When considering the use of coercive measures in the inpatient setting (mechanical restraint and isolation), data showed a significant reduction of the number of patients, who experienced at least one coercive measure. When analyzing the two forms of coercive measures separately, results showed that the number of patients subjected to restraint and the number of restraint events were reduced, although that this reduction only reached significance in the acute wards subsample. No significant reduction of the use of seclusion could be shown. Similarly to the results regarding detentions, most patients, who had experienced coercive measures during hospital stays before their inclusion in the model project, were not again subjected to such a measure during subsequent hospital stays. Thus, the model project might also have a preventive effect for the experience of coercion in inpatient settings. Restraint and seclusion episodes take place in the context of involuntary admissions and most often at the time of admission itself. Therefore, the reduction of the number of legal detentions can partly explain this preventive effect. The outpatient management of crises surely contributes to this effect, allowing patients to seek inpatient care when absolutely indicated and based on a continuous and strong therapeutic relationship. On the other hand, results show that the model project failed to prevent some of the most severe crises, particularly when patients had not experienced similar situations before. Furthermore, the relatively small effect of the model project on the use of restraint and seclusion in the inpatient setting indicates that outpatient models may only marginally influence inpatient care. Of note, the use of coercive measures is not only influenced by patient-related factors, but also by staff- and ward-related issues that cannot always be controlled by the outpatient sector (25). Moreover, the past experiences of patients with acute ward treatments and the psychiatric system in general may also play a central role in the use of coercive interventions.

Beyond the organizational factors mentioned above (22), flexibility and the long-term duration of treatment, the quality of specific therapeutic interventions offered by the model project may play a central role in the positive effects it led to. The orientation on recovery and open-dialog, as well as its strong focus on psychotherapeutic interventions, the encouragement of individual psychotherapy and the provision of treatment for co-morbid substance use disorders may also be decisive factors in its ability to adequately manage crises and prevent involuntary admissions. In contrast, Johnson et al. argued that ICM should be considered as a way of organizing teams, rather than a specific treatment model (26). Hence, the contradictory results of studies investigating the effect of ICM/ACT on coercive measures and compulsory detentions should be analyzed in the light of organizational structures as well as the specific therapeutic interventions the services deliver.

Important limitations of this study lie in its naturalistic design, the lack of a control group and the sole use of secondary data over a relatively short period of time. Findings are based on individual pre- post-comparisons and therefore cannot address the question whether the project is superior to alternative treatment approaches or solve the problem of regression to the mean. Furthermore, the influence of independent longitudinal changes within the psychiatric support system cannot be excluded. However, the majority of patients in our study had been in contact to the in- and outpatient psychiatric facilities of the hospital even longer than the 2 years prior to inclusion to the project, when detentions and coercive measures obviously could not be prevented as effectively. Moreover, effects of medication changes like switching to long-acting injectable antipsychotics on hospital bed use and coercive interventions were not investigated. As treatment in the model project fosters participation in treatment decisions and rather facilitates dose-reductions in individual patients, it can be assumed that results did not come at the price of systematic increases in outpatient medication.

Strengths of the study are the complete inclusion of all patients of one insurance company to the model project as well as in the analysis, resulting in an unselected patient sample. Equally, both the regional legal mandate of the hospital with a defined catchment area and the structure of the model project did not allow a selection of “responders”, and the assertive structure might rather have facilitated therapeutic contact with patients who are usually hard to reach (12).

In summary, our results indicate a potential of flexible, multiprofessional, outreaching treatment models that act on a need-adapted basis to reduce involuntary hospital treatments and coercive interventions in psychiatry. Findings must be substantiated by evidence from randomized-controlled trials and by studies allowing for longer observation periods.

## Author contributions

AW contributed to study design, data collection and analysis and to the main part of the manuscript redaction; JB contributed to data collection and analysis; FB supervised the research process and the manuscript redaction; CM designed the study, contributed to data analysis and redaction of the manuscript.

### Conflict of interest statement

The authors declare that the research was conducted in the absence of any commercial or financial relationships that could be construed as a potential conflict of interest.
